# Fixation of pelvic acetabular fractures using 3D-printed fracture plates: a cadaver study

**DOI:** 10.1186/s13018-023-03756-y

**Published:** 2023-05-16

**Authors:** Dale L. Robinson, Andrew Bucknill, Alessandro Ferragina, Christopher Campbell, Peter Vee Sin Lee

**Affiliations:** 1 Department of Biomedical Engineering, University of Melbourne, Melbourne, Australia; 2 Department of Surgery, University of Melbourne, Melbourne, Australia; 3 Department of Orthopaedic Surgery, Royal Melbourne Hospital, Parkville, Australia; 4 DePuy Synthes Companies, Warsaw, USA

**Keywords:** Pelvis, Fractures, 3D-printing, Additive manufacturing, Screw angulation

## Abstract

Open reduction and internal fixation of pelvic acetabular fractures are challenging due to the limited surgical exposure from surrounding abdominal tissue. There have been a number of recent trials using metallic 3D-printed pelvic fracture plates to simplify and improve various elements of these fracture fixation surgeries; however, the amount of time and accuracy involved in the design and implantation of customised plates have not been well characterised. This study recorded the amount of time related to the design, manufacture and implantation of six customised fracture plates for five cadaveric pelvic specimens with acetabular fracture, while manufacturing, and surgical accuracy was calculated from computed tomography imaging. Five of the fracture plates were designed within 9.5 h, while the plate for a pelvis with a pre-existing fracture plate took considerably longer (20.2 h). Manufacturing comprised 3D-printing the plates in Ti6Al4V with a sintered laser melting (SLM) 3D-printer and post-processing (heat treatment, smoothing, tapping threads). The manufacturing times varied from 27.0 to 32.5 h, with longer times related to machining a thread for locking-head screws with a multi-axis computer numerical control (CNC) mill. For the surface of the plate in contact with the bone, the root-mean-square errors of the print varied from 0.10 to 0.49 mm. The upper range of these errors was likely the result of plate designs that were relatively long with thin cross-sections, a combination that gives rise to high thermal stresses when using a SLM 3D-printer. A number of approaches were explored to control the trajectories of locking or non-locking head screws including guides, printed threads or hand-taps; however, the plate with CNC-machined threads was clearly the most accurate with screw angulation errors of 2.77° (range 1.05–6.34°). The implanted position of the plates was determined visually; however, the limited surgical exposure and lack of intra-operative fluoroscopy in the laboratory led to high inaccuracies (translational errors of 1.74–13.00 mm). Plate mal-positioning would lead to increased risk of surgical injury due to misplaced screws; hence, it is recommended that technologies that can control plate positioning such as fluoroscopy or alignment guides need to be implemented into customised plate design and implantation workflow. Due to the plate misalignment and the severe nature of some acetabular fractures comprising numerous small bone fragments, the acetabular reduction exceeded the clinical limit of 2 mm for three pelvises. Although our results indicate that customised plates are unsuitable for acetabular fractures comprising six or more fragments, confirmation of this finding with a greater number of specimens is recommended. The times, accuracy and suggested improvements in the current study may be used to guide future workflows aimed at producing customised pelvic fracture plates for greater numbers of patients.

**Supplementary Information:**

The online version contains supplementary material available at 10.1186/s13018-023-03756-y.

## Introduction

Fractures of the pelvic ring and/or acetabulum represent 1.5% of all fracture cases [1]; however, they have a high mortality of 7 to 30% [2] that results from secondary injures to the adjacent organs, nerves and blood vessels. In many cases, these high-risk fractures must be treated by open reduction and internal fixation; however, due to the relative rarity in pelvic fractures and anatomical variability, there is a paucity of anatomical fracture plates available for this bodily location. Straight or curved fracture plates are commonly used to treat pelvic fractures; however, these must be contoured to fit fractured bone, which weakens the material and is both time-consuming and imprecise, especially given the restricted surgical access imposed by the abdominal organs and tissues. Moreover, these off-the-shelf plates and associated instruments are unable to locate screw trajectories relative to the bone; hence, fluoroscopy or surgical navigation is required to monitor screw trajectories, which takes additional time to manage intra-operatively.

To overcome the issues related to off-the-shelf plates, there is a growing interest in the use of 3D-printed customised pelvic fracture plates. This patient-specific approach creates fracture plates that conform to the surface of the bone and can incorporate design features to control the screw trajectories. Without needing to contour the plate or implement screw trajectories intra-operatively, there have been clear benefits in surgical time observed in previous trials comparing customised to standard pelvic fracture plates [3]. Additional advantages were also highlighted in a recent systematic review and meta-analysis of trials using 3D-printed pelvic fracture plates, showing that the personalised approach yielded significant improvements in the inter-operative blood loss, post-operative complications, the extent of fracture reduction and pelvic function, as well as operating time [4].

Despite the promising clinical results with 3D-printed pelvic fracture plates, the timeframes involved in the design and manufacture of personalised fracture plates has been loosely defined in most previous trials, where total durations of 3 to 4 days were quoted [3, 5, 6], with little specificity provided on the timing of individual steps. Moreover, little information has been provided regarding the accuracy of the manufacturing, plate positioning and screw angulation. Detailed knowledge of the times involved in the design and manufacture is essential for evaluating how the 3D-printed fracture plate workflow may be further developed to meet the short timeframes between injury and operative treatment, as well as the feasibility of using this personalised approach for a greater number of patients. Understanding the accuracy is also important given the potential for mal-aligned components to cause injury to surrounding anatomical structures.

This study aims to identify the timing and accuracy involved in the use of 3D-printed pelvic fracture plates. An acetabular fracture was created in a series of cadaveric pelvis, from which the timing of each step involved in the design, manufacture and implantation was recorded. A range of different approaches were considered for controlling screws trajectories, with differences in surgical useability recorded. Finally, the accuracy of the plate manufacturing, and the surgical plate and screw positioning were quantified for each cadaver using computed tomography (CT). It was envisaged that these data will provide a greater knowledge of the resources and underlying accuracy related to the use of 3D-printed pelvic fracture plates, which will better inform related cost/benefit analyses and establish key improvements needed for using this customised approach on greater number of patients.

## Methods

### Specimens and fracture

Five fresh-frozen cadaveric pelvic specimens (M/F: 1/4, age range 66–95 years) were sourced from the University of Melbourne body donor program with ethical approval (ID: 1852689). The pelvic region was dismembered from the respective donor by axially sectioning all tissue midway through the lumbar spine and femurs, with all tissues surrounding the pelvis left intact. Each of these specimens was held in a lateral position by a clamping fixture, with the left side of three specimens facing upwards and the right side of the remaining two specimens facing upwards. An orthopaedic surgeon (AB) exposed the upper acetabulum via a Kocher–Langenbeck approach, and the femoral head was dislocated from the joint. A small acetabular fracture was initiated by impacting a chisel with a hammer into a carefully selected part of the lunate surface. The extent of this fracture was increased by impacting a ball-ended punch with a similar diameter to the femoral head. The direction of impaction was oriented to be similar to that expected by the femur such that the injury would match common fracture patterns seen at the pelvis. Following the fracture, the femoral head was return to its native position, and the incision sutured to reduce ingress of air into the joint.

### Imaging

A pre-operative CT scan was obtained based on a clinical imaging sequence to ensure the plate design represented a realistic image resolution (Table 1). A post-operative CT scan was also obtained; however, its purpose was only for determining accuracy, which is not typically assessed with CT in clinical settings; hence, the scan resolution was set as high as possible irrespective of the radiation dosage (Table 1). Once scanned, all bony anatomy including individual bone fragments was segmented with Mimics (version 21.0; Materialise, Leuven, Belgium) by the following steps in the software:

- Creating a mask for bone by using *Threshold* with the default Hounsfield Units (HUs) for bone (226 -3071 HU),
- Removing islands of bone by using the *Region Grow* with 6-connectivity,
- Removing the sacrum and contralateral hemipelvis and cleaning the boundary of the ipsilateral hemipelvis by tracing out their boundaries with *Multiple Slice Edit*,
- Filling the interior of the hemipelvis using *Smart Fill*,
- Removing fracture lines from the mask using *Multiple Slice Edit,*
- Separating the bone fragments using the *Split Mask*,
- Removing spikes for each mask using *Smooth Mask*,
- Converting the masks to 3D parts using *New Part* set at optimal quality,
- Cleaning each 3D part using *Smooth* with 10 iterations and a smooth factor of 0.5,
- Removing noise and small gaps using *Wrap* with smallest detail 0.5 mm and gap closing distance of 1 mm.

**Table 1:** CT acquisition settings used to image fracture pelvis

Parameter	Pre-operative scan	Post-operative scan
Slice thickness (mm)	0.60–0.80	0.60
Slice increment (mm)	0.50–0.60	0.10–0.30
In-plane resolution (mm)	0.48–0.74	0.74–0.76
Current (mA)	18–19	342–552
Voltage (kV)	100	120–140
Exposure (ms)	1000	1000–1558

The fracture was virtually reduced by rotating and translating each displaced bone fragment until the surface of the hemipelvis appeared smooth and congruent. The more challenging reductions utilised the mirrored contralateral hemipelvis to guide the alignment.

### Design workflow

**Fig. 1:**
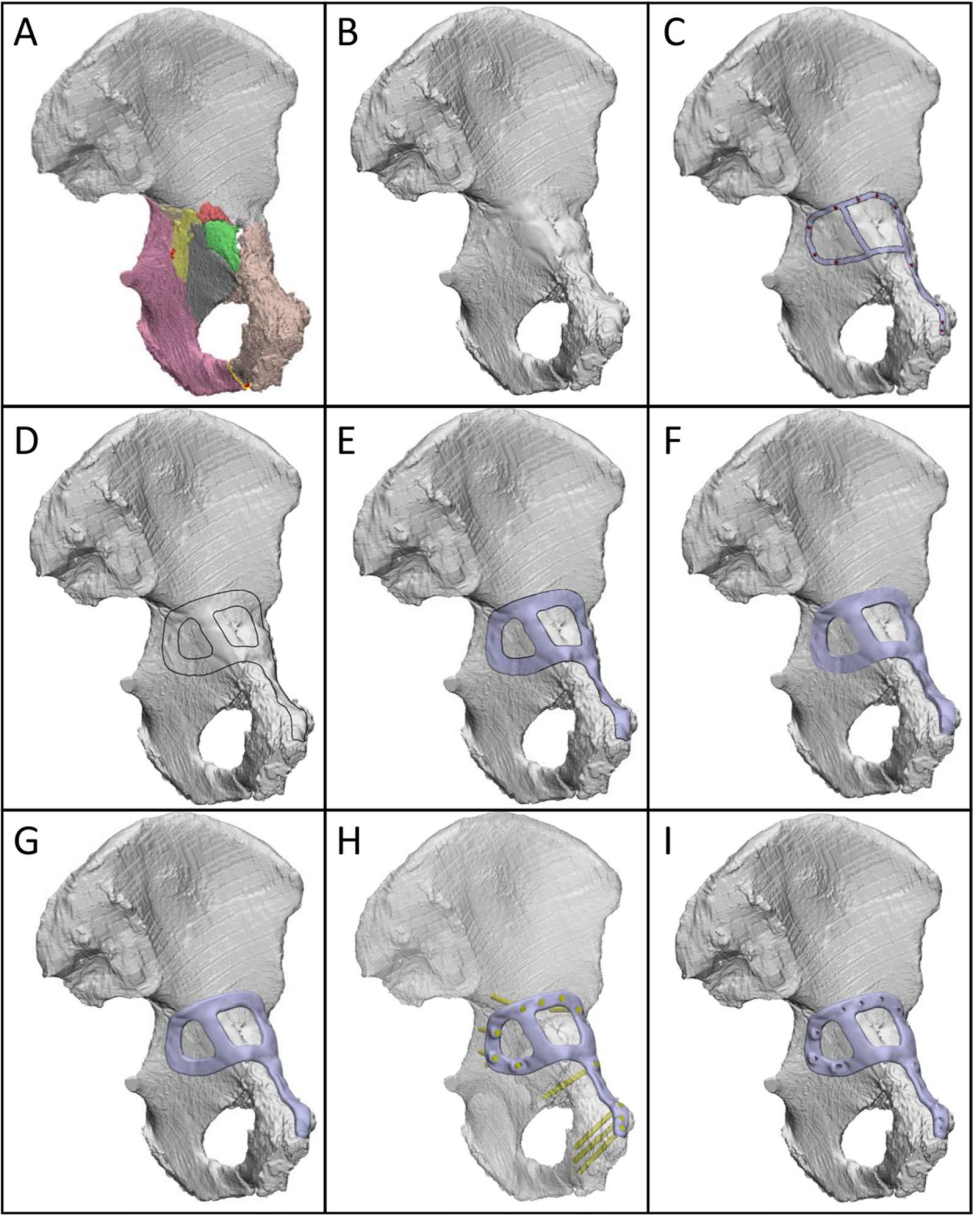
Design workflow for creating customised fracture plates. **A** Left hemipelvis with reduced fracture. Each piece of bone is indicated by a different colour. **B** Hemipelvis (grey) with fracture lines closed. **C** Intended path of plate (silver) and screw positions (red). **D** Curves indicating plate outline. **E** Plate lower surface, offset above bone by 0.2 mm. **F** Extruded plate geometry. **G** Plate geometry with filleted edges.** H** Plate and trajectories of 3.5 mm bicortical screws (yellow cylinders). The hemipelvis is displayed as transparent for visualisation. **I** Plate with holes cut through by screw cylinders

Once the fracture was reduced (Fig. 1A), the design workflow for each customised plate comprised the following steps, which were performed using either of the commercial software packages Geomagic Freeform Plus (version 2019.2.50; 3D systems, Bethesda, USA), Geomagic Wrap (version 2015; 3D systems, Bethesda, USA) and Ossa 3D (Conceptualiz, Toronto, Canada):

1. All fracture lines were closed with a fill option (Fig. 1B).
2. An orthopaedic surgeon marked out the intended plate path and screw locations using a highlighting tool in the software (Fig. 1C).
3. Curves were drawn to follow the surgeons' path based on a constant plate width (Fig. 1D). The plate width for each case varied between 8 and 12 mm, and the curves were smoothed to avoid sharp edges.
4. The curves were used to cut the bone, thus creating the lower surface of the plate. This surface was offset by 0.2 mm above the bone surface to allow clearance for the periosteal surface of the bone and defects in manufacturing (Fig. 1E).
5. The lower surface of the plate was extruded to a thickness of 3 to 3.5 mm (Fig. 1F).
6. The edges of the plate were filleted to avoid sharp edges (Fig. 1G).
7. Based on the screw positions indicated by the surgeon, trajectories for 3.5-mm bicortical screws were defined using cylinders of identical diameter (Fig. 1H). The trajectories were chosen based on the following constraints or requirements:
  Maximise the length of bone contacting the screw,
  Avoid critical anatomy such as the acetabular surface,
  For their trajectory to be accessible through the surgical windows,
  For screws initiating from the medial surface of the pelvis, place 2 or 3 screws across the roof of the acetabular dome (i.e. rafting screws) and
  Place 2 or 3 screws through the pubic symphysis region.
8. Finally, the head of the screw was cut into the plate (Fig. 1I).

Plates were designed to use either locking or variable angle (VA) non-locking screws. In the case of locking screws, the threads for the respective heads were either printed directly into the plate or were machined by a 5-axis computer numerical control (CNC) mill. For VA screws, a number of different methods were trailed to control screw trajectories, which are outlined in the results section for the applicable cases. The plate thickness was chosen based on commercially available fracture plates such as the DePuy Synthes low profile pelvic fracture plate (3.5 mm thickness), the Stryker Matta fracture plate (3.2 mm thickness) or the Acumed reconstruction plate (3.5 mm thickness) and is consistent with the previous work using customised pelvic fracture plates [6]. To confirm the assumed thickness provided sufficient strength, a finite element analysis was performed for fracture Case 1 (see Additional file 1). The maximum von Mises stress of the fracture plates was 169.2 MPa, which is considerably lower than the yield strength reported for Ti6Al4V alloys fabricated by 3D-printers with annealing of 741–1045 MPa [7].

### 3D-printing and post-processing

The plates were 3D-printed in Ti6Al4V using a sintered laser melting (SLM) printer (Renishaw AM250 or 3D Systems DMP Prox 320) with a 30-µm slice thickness. Each plate was positioned for printing based on a trade-off between reducing the vertical height to reduce build time, using the minimum amount of support material to reduce post-processing smoothing tasks yet provide sufficient stiffness to resist thermal stress, and a preference for support material extending from the dorsal side of the plate to ensure that the ventral plate surface contacting bone did not require the removal of support material that would compromise its accuracy. The long length and thin cross-sections of the plate design were likely to cause high-temperature gradients, resulting in residual stresses; hence, a stress relief anneal was applied comprising 1 h at 538–649 °C, followed by air or furnace cooling for 2 h. Once cooled, the plate plus support material was cut from the build platform with a wire cutter; then, the support material removed with plyers. Any residual support material was removed with a sander, followed by a final clean with a bead blaster.

### Implantation

The implantation comprised either an anterior intra-pelvic approach or a posterior approach to the acetabulum, depending on the injury pattern and consequent plate design. The fractures were reduced using standard surgical reduction instruments (DePuy Synthes); then, plates were positioned in a two-step process. Firstly, the plate was roughly placed in the targeted position based on a qualitative comparison of its planned alignment relative to visual landmarks. For plates implanted via the anterior intra-pelvic approach, the landmarks included the pubic symphysis (midline), pubis tubercle, pelvic brim, obturator foramen and the sciatic notch, whereas for posterior pelvic approaches the landmarks were the ischial tuberosity, posterior wall and sciatic notch. Secondly, minor repositioning of the plate was performed until the plate appeared and felt congruent with the underlying bone. Screws were implanted through the plates as per the pre-operative surgical plan and design.

### Accuracy and timing

To assess how accurately the bones and plates were positioned compared to what was planned, the bone, plate(s) and screws were segmented from the post-operative CT images and converted to 3D objects using Mimics. The rigid body transformation matrix was computed by an iterative closest point (ICP) algorithm in Geomagic to register the large post-operative bone fragment comprising the ilium to the pre-operative hemipelvis. This transformation matrix was subsequently applied to all remaining post-operative bone fragments, plates and screws, thus establishing global registration between the pre- and post-operative hemipelvis. The difference between the pre- and post-operative positions of individual components was computed by respective ICP registrations, from which the resultant translation and rotation were calculated for the plates (*T*_plate_ and *R*_plate_, respectively) and the bones (*T*_bone_ and *R*_bone_, respectively). The mismatch between the acetabula was computed by the root-mean-square (RMS) deviation between the pre- and post-operative acetabular surfaces (*RMS*_acet_). The difference in the screw trajectories was computed by aligning the designed and post-operative fracture plate geometries and computing the angular difference between the respective screw shaft vectors (*θ*_screw_).

To determine the manufacturing accuracy, each printed plate was scanned with a micro-CT scanner (Phoenix nanotom m; GE, Boston, USA). The fracture plate manufacturing accuracy was computed by the RMS deviation between the designed surface of the fracture plate and its surface determined from the post-operative CT. This RMS deviation was computed for both the entire surface of the plate (*RMS*_plate_all_) and its ventral surface contacting the bone (*RMS*_plate_bone_). In either case, the screw–head interface was not included in the calculation.

To analyse which variables influenced the bone alignment, independent stepwise multiple linear regressions were performed in SPSS (version 29, SPSS Inc., Chicago, IL, USA) with the dependent variables: *T*_bone_, *R*_bone_ and *RMS*_acet_, and the independent predictor variables: number of bone fragments (*N*_frag_), *RMS*_plate_all_, *RMS*_plate_bone_, *T*_plate_, *R*_plate_ and *θ*_screw_. This stepwise approach sequentially includes predictors if the corresponding fit of the regression is significantly improved according to a partial F-test, whilst predictors that do not satisfy this criterion at any point in the regression are eliminated. The significance of the final regression is also assessed using an F-test. The level of significance for all analyses was set as *p* < 0.05.

The timing was recorded, respectively, for CT image processing (segmentation and fracture reduction), plate design, screw design, alignment guide design, 3D-printing, post-processing and implantation. The 3D-printing included the software set-up for the print, preparing the printing equipment (wiper, lens and laser), 3D-printing the part and cooling of the part. Post-processing comprised thermal annealing, support material removal with a wire cutter and pliers, smoothing the part with a sander and bead blasting and, where applicable, machining of screw threads.

## Results

### Case 1 design and implantation

**Fig. 2:**
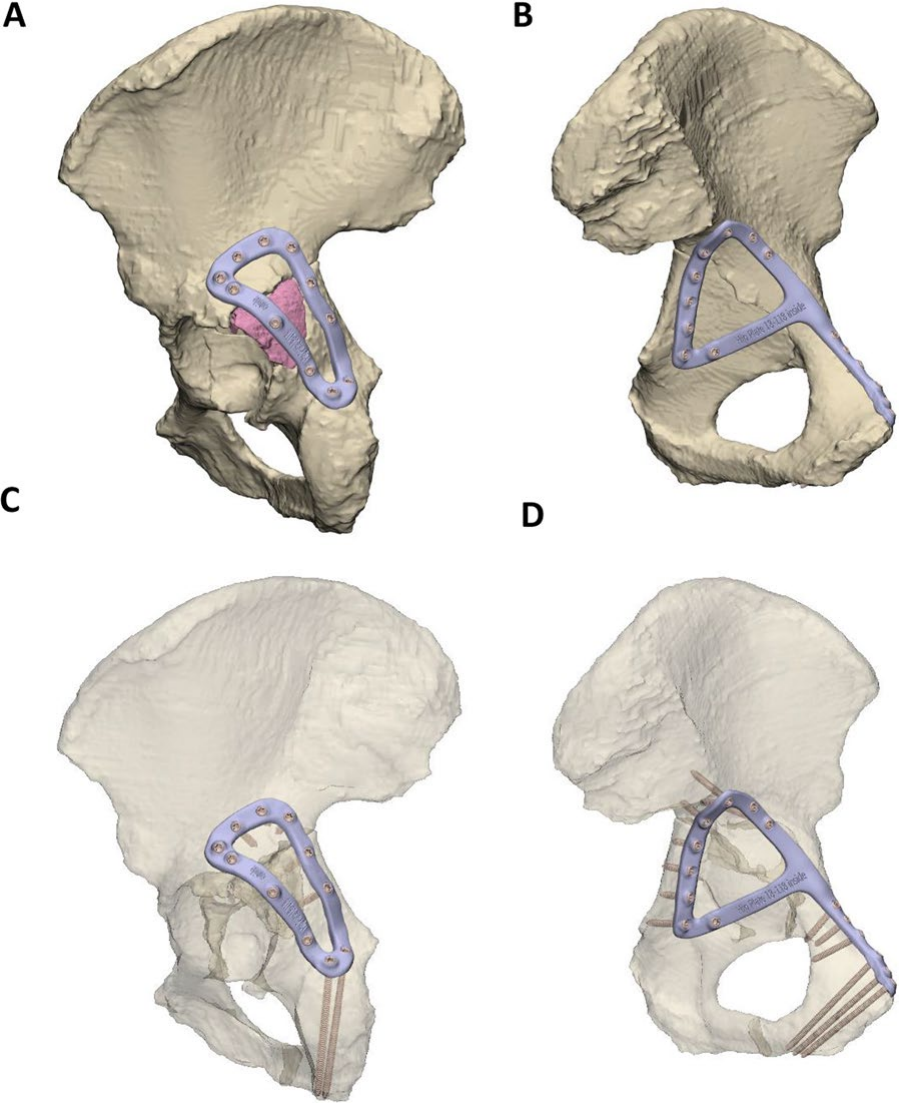
Case 1** l**eft hemipelvis and fracture plate design. **A** Lateral view of the reduced hemipelvis with a bone fragment indicated in pink and the lateral fracture plate shown. **B** Medial view of hemipelvis with the central dislocation fracture visible and the medial fracture plate shown. The trajectories of the screws are shown for the lateral **C** and medial plate **D** with the bony geometry shown as transparent

**Fig. 3:**
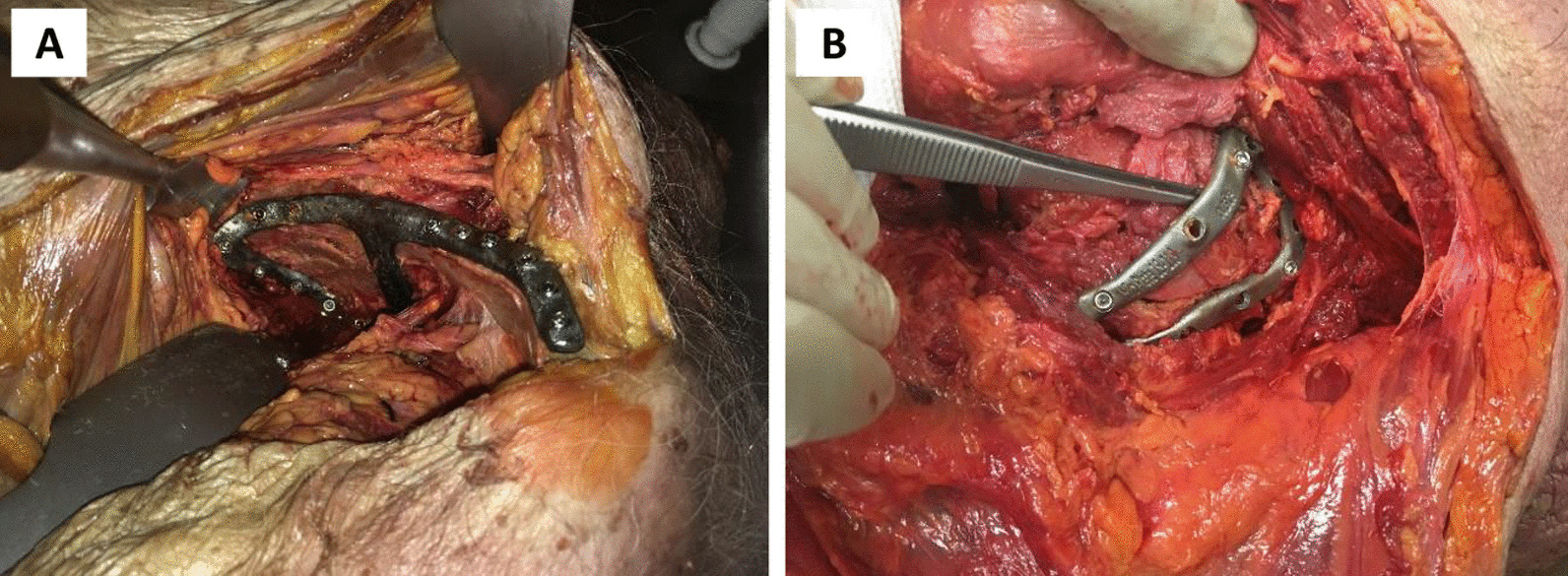
Photographs of case 1 implantation. The medial and lateral plates are shown in **A** and **B**, respectively

A transverse with posterior wall fracture of the left acetabulum was created in the cadaveric pelvis of a 95-year-old female, which resulted in displaced bone fragments on the medial and posterior aspects of the acetabulum (Fig. 2). A ‘posterior’ fracture plate with a triangular shape was designed on the posterior wall and posterior column (the dorsal surface of the ischium and the gluteal surface of the ilium) (Fig. 2A), and a ‘medial’ fracture plate was designed that extended from the pubic tubercle, along the iliopubic brim to describe a closed loop on the quadrilateral plate surface (Fig. 2B). The posterior and medial plates comprised 11 and 15 locking screws, respectively, with the mating thread for the screw head printed directly into the plate. The medial plate was first implanted via an anterior intra-pelvic approach, followed by the posterior plate via a posterior approach (Fig. 3). All screws were placed without apparent contact against one-another, however, for a number of screws it appeared that the 3D-printed threads on the plates stripped or did not engage with the threads indicating poor strength and/or print accuracy in this region.

### Case 2 design and implantation

**Fig. 4:**
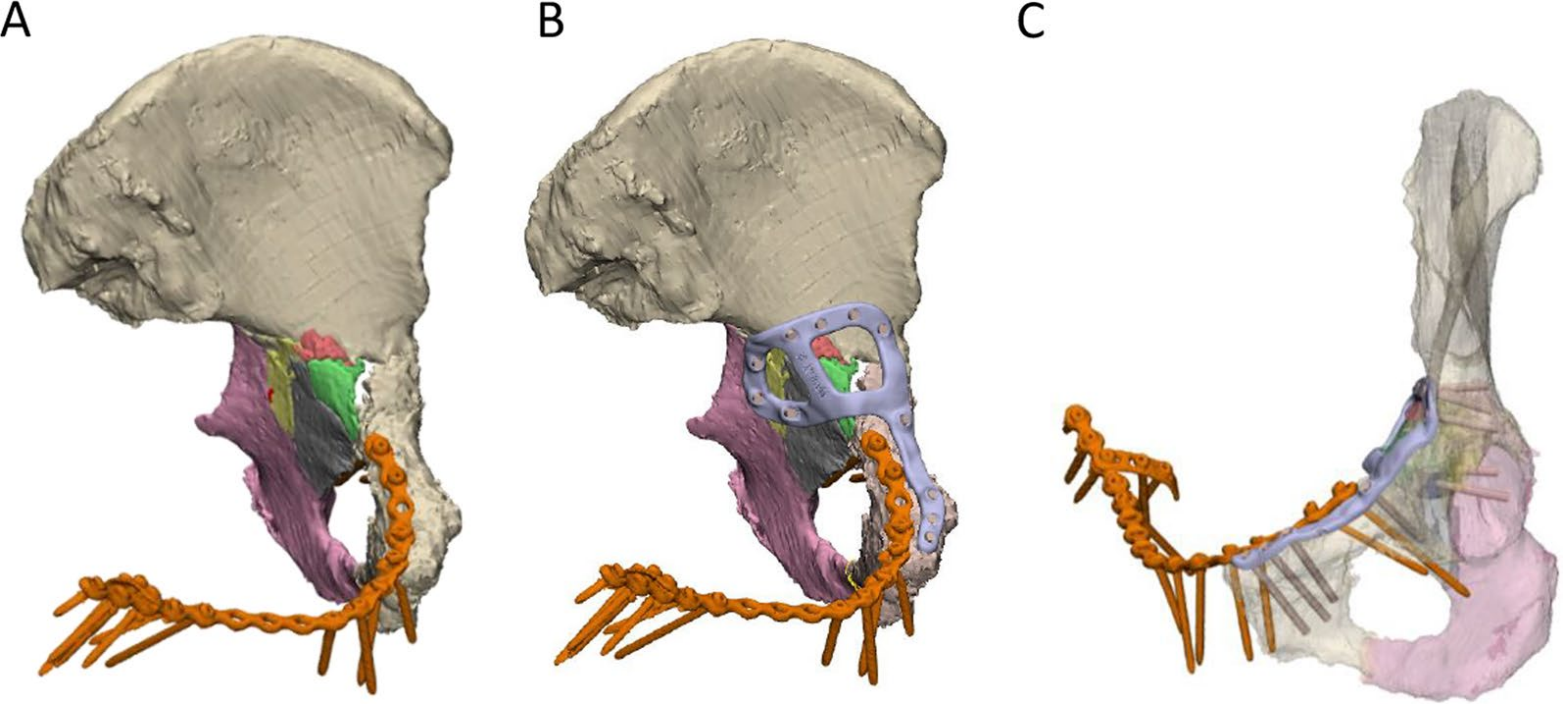
Case 2** l**eft hemipelvis and plate design. **A** The reconstructed pelvis comprising 8 fractured pieces of bone highlighted in different colours. An existing fracture plate is shown in orange. **B** Reconstructed pelvis with existing plate and customised fracture plate shown in orange and light purple, respectively. **C** The trajectories of the two plates are shown with the bone set as transparent

An anterior with posterior hemi-transverse fracture in the left acetabulum was created in the cadaveric pelvis of a 93-year-old female, which resulted in 8 fragments of bone (Fig. 4A). Notably, the pelvis had a pre-existing fracture plate extending across the iliopubic ramus of the left and right hemipelvis. A customised fracture plate was designed on the medial surface of the hemipelvis that was similar to Case 1, where it extended from the pubic tubercle, along the iliopubic brim to then describe a closed loop on the quadrilateral surface (Fig. 4B). Additionally, this plate was designed to avoid the existing fracture plate and had a cross-link added to the plate to help brace the fragmented pieces of bone. The plate comprised 11 bicortical locking screws with the thread printed directly into the plate (Fig. 4C). The plate was placed via modified stoppa approach; however, a number of the 3D-printed screw threads were observed to strip. Holding the plate with a ball spike was also observed to be challenging when placing some of the screws. Hence, it was suggested that the inclusion of small cylindrical holes in regions of the plate without screws would be useful for placing the ball spike and securing the plate.

### Case 3 design and implantation

**Fig. 5:**
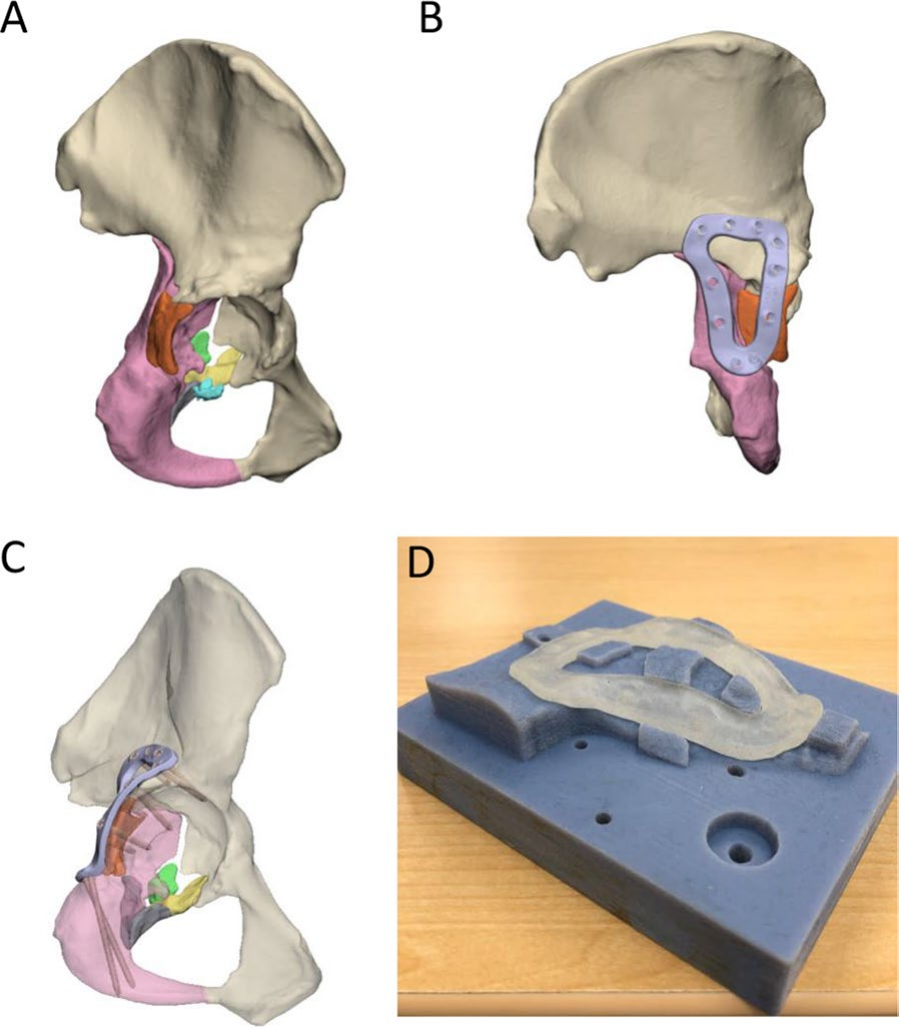
Case 3 right hemipelvis plate and clamping fixture design. **A** The reconstructed pelvis comprising 8 fractured pieces of bone highlighted in different colours. **B** Reconstructed pelvis with customised fracture plate shown in light purple. **C** The trajectories of the customised plate are shown with the bone set as transparent. **D** Customised clamping fixture used to hold the plate in a controlled position during milling. A transparent resin copy of the fracture plate is placed in position on the fixture

A posterior wall and column fracture of the right acetabulum was created in the cadaveric pelvis of a 66-year-old female, which resulted in 8 bone fragments (Fig. 5A). Notably for this case, there were still some intra-articular fracture gaps that remained following the virtual reduction, which may be the result of permanent deformation of the bone larger fragments, causing them to change shape and, therefore, making it impossible to perfectly piece the fragments back together via rigid-body translations and rotations. A fracture plate was designed on the posterior surface of the acetabulum that was similar to Case 1, bridging the posterior column and posterior wall (looped across the dorsal surface of the ischium and the gluteal surface of the ilium) (Fig. 5B) and only one design iteration was required for the plate. The plate comprised a total of 10 bicortical locking screws (Fig. 5C). The threads for the screws were machined into the plate with a 5-axis CNC mill, with a customised clamping fixture used to hold the plate in a controlled position during machining (Fig. 5D). No screw threads stripped during the implantation.

### Case 4 design and implantation

**Fig. 6:**
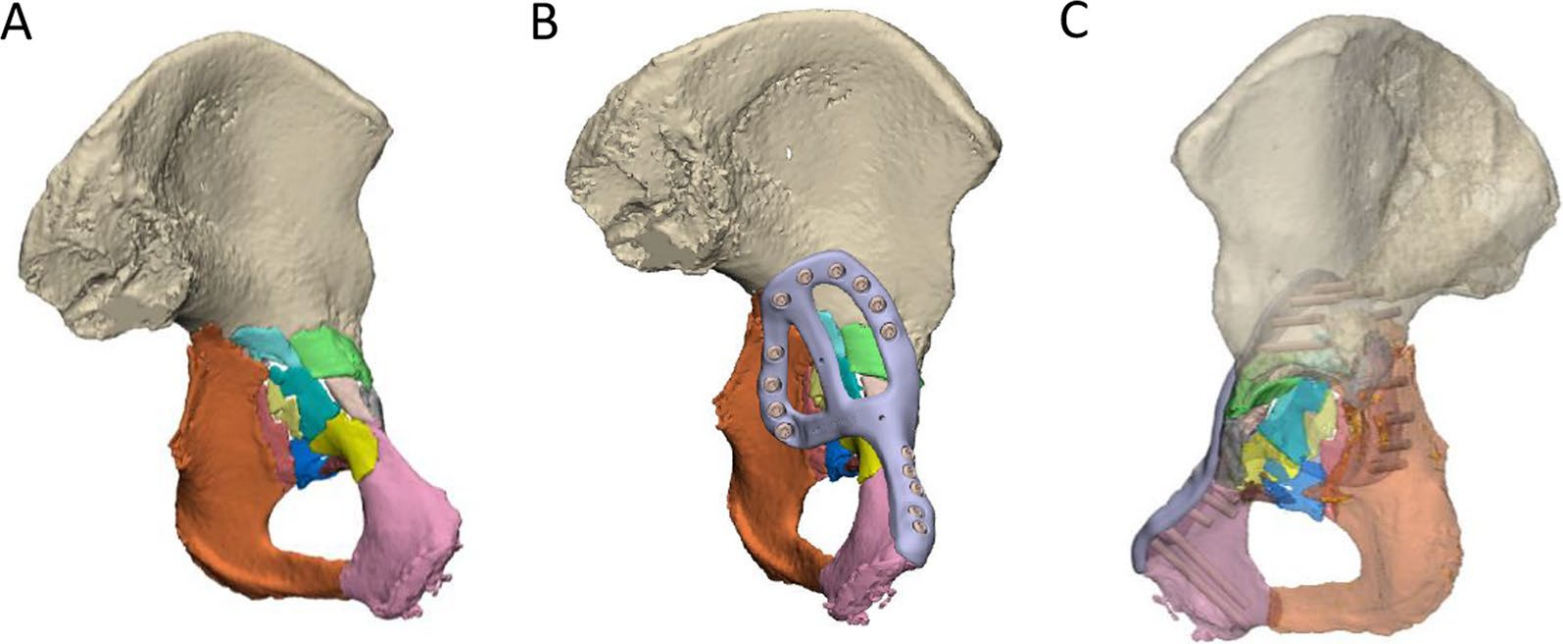
Case 4 left hemipelvis and plate design. **A** The reconstructed pelvis comprising 14 fractured pieces of bone highlighted in different colours. **B** Reconstructed pelvis with customised fracture plate shown in light purple. **C** The trajectories of the customised plate are shown with the bone set as transparent

**Fig. 7:**
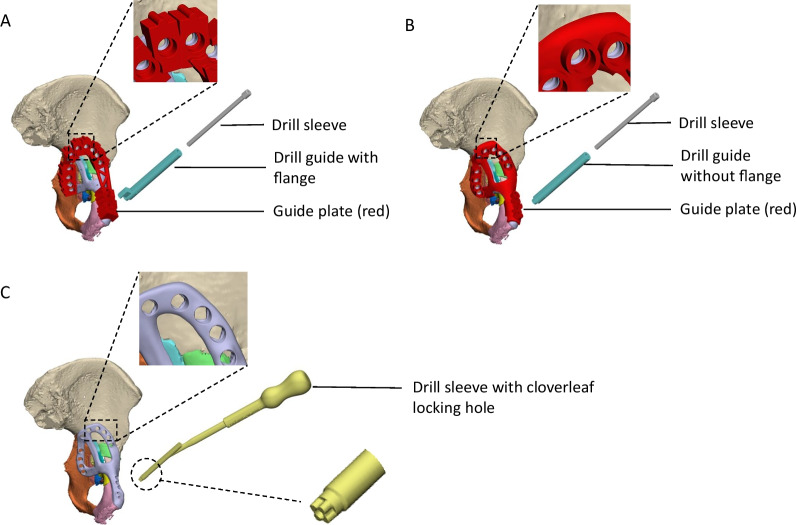
Case 4 considered options for drill guide design. **A** The first option comprised a drill guide with a flange that interfaced on a guide plate. **B** For the second option, the flange was removed from the drill guide and the shaft directly interfaced with the guide plate via a cylindrical locking hole. **C** The third option comprised a drill sleeve that interfaced directly with the fracture plate via a cloverleaf locking hole

An anterior with posterior hemi-transverse fracture of the left acetabulum was created in the cadaveric pelvis of a 91-year-old female, which resulted in 14 bone fragments (Fig. 6A). A fracture plate was designed on the medial surface of the acetabulum that was similar to that created for Case 2 (Fig. 6B). The plate comprised a total of 15 non-locking screws (Fig. 6C). To control the trajectory of these screws, three iterations of guide plates and drill guides were considered:

- The first iteration comprised a guide plate and a drill guide with a flange (Fig. 7A). The guide plate clipped onto the fracture plate and the drill guide was secured at each screw location via its cylindrical shaft and contact with the flange. The surgeon advised that this guide plate design was too bulky for the surgical exposure, and the drill guide did not appear to need a flange in order to secure it.
- In the second iteration, the flange was removed from the drill guide, which led to guide plate with a lower profile (Fig. 7B). Although the drill guide was adequately secured, the surgeon felt that this guide plate was still too bulky to fit inside the surgical exposure.
- In the third iteration, no guide plate was used and the drill sleeve interfaced directly with the fracture plate (Fig. 7C). A cloverleaf locking hole design was used to secure the drill sleeve in position on the fracture plate.

The third concept was trialled during the implantation on the fracture plate. However, even with the drill sleeve interfacing via the cloverleaf locking hole, it still had considerable play relative to the plate, suggesting that insufficient interference was present to control the angulation of the drill sleeve.

### Case 5 design and implantation

**Fig. 8:**
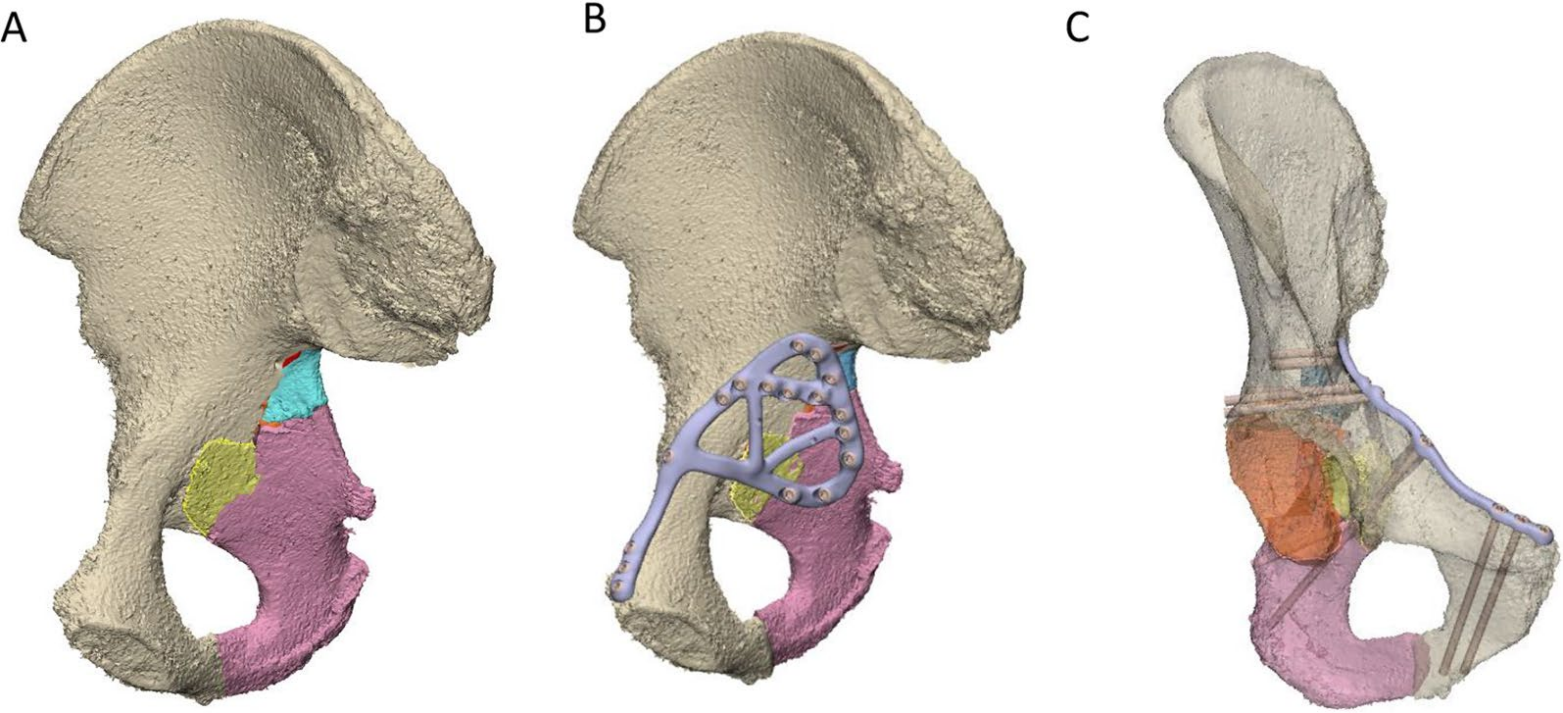
Case 5 right hemipelvis and plate design. **A** The reconstructed pelvis comprising 5 fractured pieces of bone highlighted in different colours. **B** Reconstructed pelvis with customised fracture plate shown in light purple. **C** The trajectories of the customised plate are shown with the bone set as transparent

**Fig. 9:**
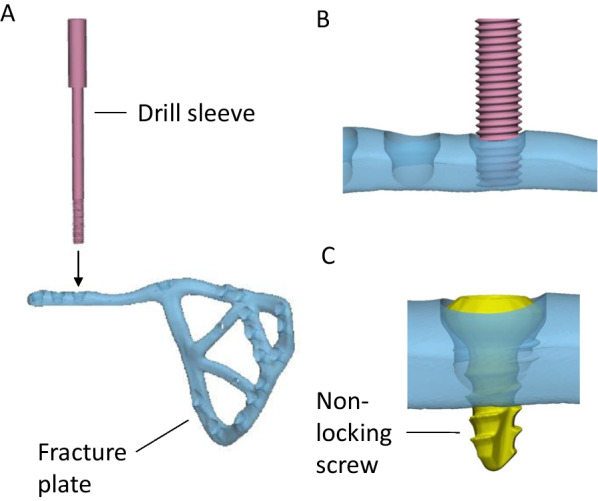
Approach for securing drill sleeve to plate using straight threads. **A** The M5 × 0.5 male thread on the drill sleeve (pink) is screwed into the female thread on the fracture plate that has been tapped by hand. **B** Drill sleeve sitting in position. **C** After the bone is drilled, the 3.5 mm non-locking is placed in the hole

**Fig. 10:**
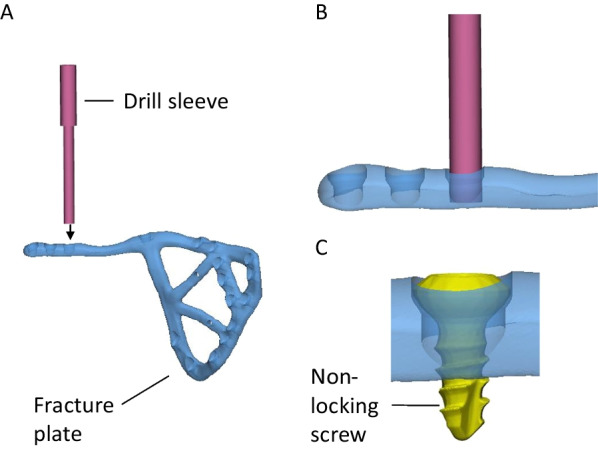
Approach for securing drill sleeve to plate using interference fit. **A** The 5-mm shaft on the drill sleeve (pink) is placed into the hole on the fracture plate that has been drilled by hand. **B** The drill sleeve is shown sitting in position. **C** After the bone is drilled, the 3.5 mm non-locking is placed in the hole

A comminuted, medially displaced posterior column fracture of the right acetabulum was created in the cadaveric pelvis of a 90-year-old male, which resulted in 5 fragments of bone (Fig. 8A). A fracture plate was designed on the medial surface of the hemipelvis that was similar to that created for Case 4; however, more cross-members were added to support the loose pieces of bone (Fig. 8B). The plate comprised a total of 17 non-locking screws (Fig. 8C). Two options were considered to secure a drill sleeve to the fracture plate:

- The first option comprised a straight M5 × 0.5 thread on the plate that engaged with a threaded drill guide (Fig. 9). For each screw position, a pilot hole of 4.5 mm with a minimum length of 1 mm was designed in the fracture plate. After printing, the hole was drilled out with a hand drill using a 4.5-mm tungsten carbide drill bit. Finally, the holes were manually tapped with a tungsten carbide tap and tap wrench. When successfully tapped, the drill guide was well-secured by the plate; however, tapping the plate was extremely difficult to execute for most holes and in some cases the tap and plate cold welded, which ultimately broke the tap.
- The second option comprised an interference fit between the cylindrical shaft of the drill guide and a cylindrical hole on the plate (Fig. 10). For each screw position, a pilot hole of 4.5 mm was designed in the print of the fracture plate, which was later drilled out with a hand drill using a 5.0 mm tungsten carbide drill bit. Due to inaccuracies in the manual drilling, the drill guide had suitable clearance to slide into each hole. It was still challenging to drill out the holes using this approach, and for some screw holes the degree of interference between the drill guide and fracture plate was inadequate to control the trajectory of the drill sleeve. Nevertheless, this approach was tried to assess its overall accuracy when implanted into the cadaveric pelvis.

**Fig. 11:**
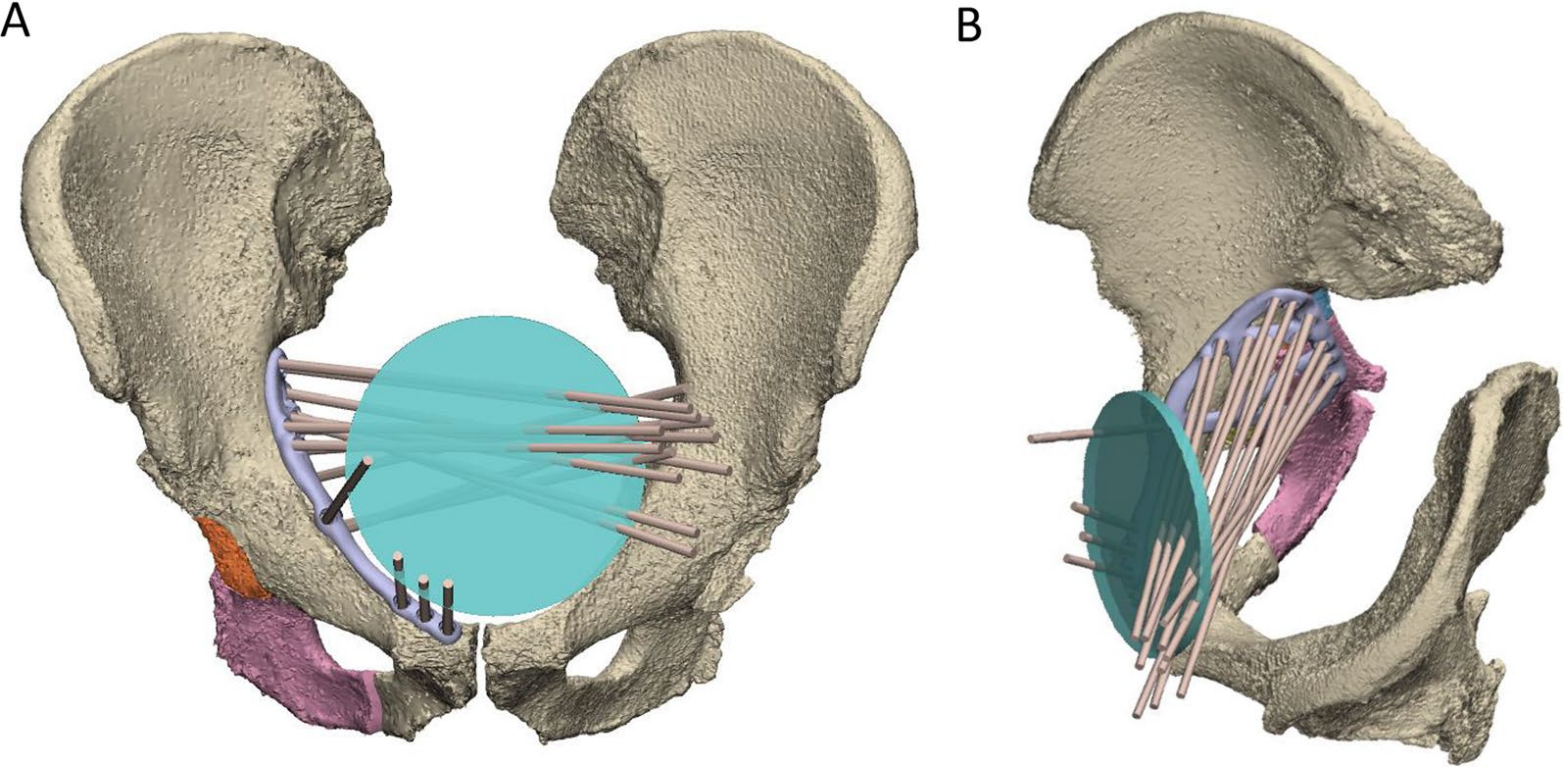
Use of 100-mm-diameter disc representing surgical window (green) to design screw trajectories. All screw trajectories (brown cylinders) were designed to pass through the disc as shown in the frontal **A** and oblique viewing planes **B**

To aid in the design of the screw trajectories, a 100-mm-diameter disc representing a surgical window for an anterior approach was placed at the midline of the suprapubic region of the abdomen (Fig. 11). A cylinder following each screw trajectory was extended anteriorly from its location on the plate to assess whether it passed through the surgical window. Each screw trajectory was iteratively adjusted to ensure all passed through the surgical window and would be surgically accessible.

### Manufacturing and surgical accuracy

**Fig. 12:**
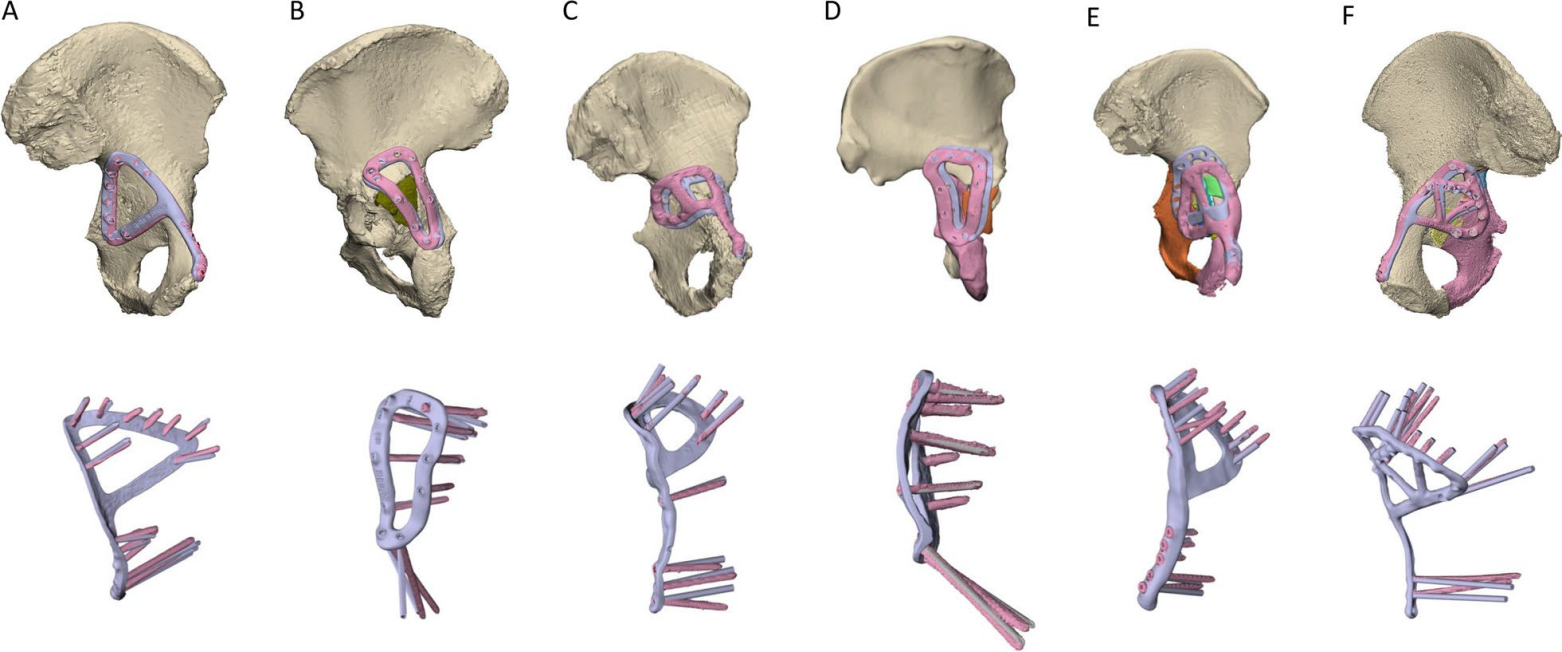
Accuracy of plate positioning and screw angulation. The top row shows the reconstructed pelvis with the customised plates in their pre-planned position (light purple) and following implantation (pink). The bottom row compares the screw angulation of the pre-planned and implanted plates, after the respective plates were rigidly aligned

In terms of the print errors across the entire plate surface, *RMS*_plate_all_ varied from 0.13 to 0.47 mm, while for the more critical ventral surface contacting the bone *RMS*_plate_bone_ varied from 0.10 to 0.49 mm (Table 2). For the plate alignment, *T*_plate_ ranged from 1.74 to 13.00 mm, and *R*_plate_, ranged from 1.58° to 8.51° (Fig. 12). *θ*_screw_ was markedly lower for Case 3, with a mean error of 2.77°. For the other plates *θ*_screw_ was 5.78–7.28°, however, there were many screws amongst these plates where the error was greater than 10° (Fig. 12). The post-operative positions of the bone fragments varied from their planned positions with T_bone_ ranging from 0.81 to 7.23 mm, and *R*_bone_ ranging from 1.13° to 17.66°. The pre- and post-operative acetabular surface variable, *RMS*_acet_, varied from 1.38 to 3.01 mm. The stepwise linear regression (Table 3) indicated *N*_frag_ was a significant predictor for both *T*_bone_ (*p* = 0.013, *R*^2^ = 0.821) and *RMS*_acet_ (*p* = 0.026, *R*^2^ = 0.748, see also Eq. 1). *R*_bone_ was significantly correlated to *T*_plate_ (*p* = 0.018, *R*^2^ = 0.792).

1 $${\text{RMS}}_{{{\text{acet}}}} = 0.{12}0*{N}_{{{\text{frag}}}} + {1}.{372}$$

**Table 2:** Print deviation, plate and screw alignment and bone fragment positioning errors

	Print deviation		Plate and screw alignment			Bone fragments			
	*RMS*_plate_all_ (mm)	*RMS*_plate_bone_ (mm)	*T*_plate_ (mm)	*R*_plate_ (°)	*θ*_screw_ mean, range (°)	*N*_frag_	*T*_bone_ mean, range (mm)	*R*_bone_ mean, range (°)	RMS_acet_ (mm)
Case 1 medial	0.28	0.29	3.54	1.58	5.78, 1.75–8.74	2	1.54, 0.81–2.27	3.68, 1.13–6.22	1.74
Case 1 lateral	0.47	0.37	1.74	4.90	6.20, 0.73–12.95				
Case 2	0.46	0.49	5.66	8.51	6.96, 2.21–14.28	8	3.81, 2.84–5.05	5.76, 3.36–12.08	2.67
Case 3	0.13	0.10	7.49	5.73	2.77, 1.05–6.34	8	4.26, 1.68–6.33	7.95, 4.05–14.25	2.36
Case 4	0.17	0.13	13.00	7.69	6.34, 1.01–20.26	14	4.76, 3.43–5.8	10.95, 2.12–19.39	3.01
Case 5	0.26	0.26	5.12	8.51	7.28, 1.11–13.28	5	3.62, 0.4–7.23	8.59, 1.81–17.66	1.38

*RMS*_*plate_all*_ root-mean-squared printing error of all plate surfaces; *RMS*_*plate_bone*_ root-mean-squared printing error of plate surface contacting bone; *T*_*plate*_ translation resultant for plate misalignment; *R*_*plate*_ rotation resultant angle for plate misalignment; *θ*_*screw*_ screw angulation error; *N*_*frag*_ total number of bone fragments; *T*_*bone*_ translation resultant for bone fragment misalignment; *R*_*bone*_ rotation resultant angle for bone fragment misalignment; *RMS*_*acet*_ root-mean-squared deviation between pre- and post-operative acetabular surfaces

**Table 3:** Stepwise multiple linear regression results describing the dependencies of the bone fragment translation and rotational errors

Outcome variable	Step	Included predictor variable	*R*^2^	B	*β*	*p*-value	Excluded predictor variables (corresponding *p*-value)
T_bone_	1	Constant	0.821	1.462		0.042	*RMS*_plate_all_ (0.644)
		N_frag_		0.275	0.906	0.013	*RMS *_plate_bone_ (0.830)
							*T *_plate_ (0.748)
							*R*_plate_ (0.268)
							*θ*_screw_ (0.767)
R_bone_	1	Constant	0.792	2.732		0.085	*N *_frag_ (0.975)
		T_plate_		0.663	0.890	0.018	*RMS*_plate_all_ (0.623)
							*RMS*_plate_bone_ (0.638)
							*R*_plate_ (0.253)
							*θ*_screw_ (0.790)
RMS_acet_	1	Constant	0.748	1.372		0.007	*RMS*_plate_all_ (0.397)
		N_frag_		0.120	0.865	0.026	*RMS*_plate_bone_ (0.429)
							*T*_plate_ (0.477)
							*R*_plate_ (0.484)
							*θ*_screw_ (0.732)

*R* Pearson’s correlation coefficient; *B* B-coefficient representing regression slope between predictor and outcome variable; *β* standardised beta-coefficient; *RMS*_*plate_all*_ root-mean-squared printing error of all plate surfaces; *RMS*_*plate_bone*_ root-mean-squared printing error of plate surface contacting bone; *T*_*plate*_ translation resultant for plate misalignment; *R*_*plate*_ rotation resultant angle for plate misalignment; *θ*_*screw*_ screw angulation error; *N*_*frag*_ total number of bone fragments; *T*_*bone*_ translation resultant for bone fragment misalignment; *R*_*bone*_ rotation resultant angle for bone fragment misalignment; *RMS*_*acet*_ root-mean-squared deviation between pre- and post-operative acetabular surfaces

### Timing

A summary of each case including the time involved in the design, printing and implantation is provided in Table 4. In general, the segmentation and reconstruction varied between 1 and 4 h, which was mostly dependent on the complexity of the fracture and how many fragments were present. Each plate required 1 or 2 design iterations, apart from the very first plate designed (Case 1 lateral) and the pelvis with a pre-existing plate (Case 2), with each requiring 3 iterations. Case 2 also took much longer than the other cases (11.7 h), which was mostly a consequence of challenges relating to excessive metal artefact noise in the CT caused by the pre-existing plate. Apart from Case 2, it was notable that the design time of the screw trajectories was equal to or greater than the design time of the plate surface, which underscores the key challenge related to trajectory design that establishes primary stability while being surgically accessible. Only Case 4 involved a screw trajectory alignment guide, which required 3 h to design and print.

**Table 4:** Case descriptive data, duration of design and print, print accuracy and positioning errors

ID	Sex (M/F)	Fracture type	Age (years)	Design iterations (#)	CT image processing (hours)	Plate design time (hours)	Screw design time (hours)	Guide design (hours)	3D-printing duration (hours)	Post-processing (hours)*	Implantation time (hours)	Total (hours)
Case 1 medial	F	Central dislocation fracture	95	2	2.5	1.5	2	0	27	5	0.8	40.8
Case 1 lateral	F	Central dislocation fracture	95	3	1	1	1.5	0	27	5	0.7	39.2
Case 2	F	Posterior wall fracture	93	3	4	11.7	1.5	0	27	5	0.8	52.2
Case 3	F	Posterior wall and column	66	1	1	1	1	0	18	14.5	0.8	37.3
Case 4	F	Transverse with central dislocation	91	2	3.5	1	2	3	27	5	0.8	44.3
Case 5	M	Posterior wall and column	90	1	3.5	1.5	2.5	0	22	5	1.0	36.5
Mean ± 1.0 STD			88.3 ± 11.1	2.0 ± 0.9	2.6 ± 1.3	3.0 ± 4.3	1.8 ± 0.5	0.5 ± 1.2	24.7 ± 3.8	6.6 ± 3.9	0.8 ± 0.1	41.7 ± 5.8

*Post-processing comprised thermal anneal, support removal, smoothing operations (bead blasting, sanding)

The plates for Cases 3 and 5 were printed with the 3D Systems DMP Prox 320 and had a print duration of 18 h and 22 h, respectively. The other four plates were each printed in 27 h; a longer duration was attributed to the use of the older Renishaw AM250 printer. Post-processing for all plates other than Case 3 took 5 h, comprising 1 h stress relief annealing, 2 h for air-cooling and 2 h for basic machining tasks related to support material and smoothing. Case 3 required 14.5 h to post-process due to an additional 9.5 h required for CNC machining mating threads for locking screws. All plates were implanted within 0.8–1.0 h.

## Discussion

Off-the-shelf fracture plates comprising simple geometric shapes are commonly used to treat pelvic acetabular fractures; however, their use is time-consuming as they must be contoured intra-operatively to match patient anatomy. Moreover, fixation screws must be carefully monitored to establish primary stability while avoiding critical anatomy. To examine a solution to these practical challenges, this study quantified the accuracy and time involved in the design, manufacture and implantation of 3D-printed acetabular fracture plates for a case series of five cadaveric pelvis. The six plates that were developed each followed one of two designs; a triangular-shaped posterior plate that looped across the posterior wall and column (Fig. 2A), or a medial plate that extended from the pubic tubercle, along the iliopubic brim to then describe a closed loop on the quadrilateral surface (Fig. 6B). Each plate was manufactured in Ti6Al4V via 3D-printing and implanted by an orthopaedic surgeon. All steps in the design, manufacture and implantation were timed, and the manufacturing and surgical accuracy were calculated via CT imaging.

As anticipated, the use of 3D-printed fracture plates led to a significant improvement in their surgical useability, where plates could be rapidly positioned without contouring, and the pre-planned screw trajectories controlled by the plate design meant that screws could be placed without intra-operative measurements or monitoring; benefits that led to implantation times of one hour or less for all fracture plates. Unfortunately, there are no implantation times currently reported for the use of conventional plates on cadaveric pelvis, however, for live patients reported implantation times were mostly higher than the current study (mean operation times of 72.29–187.47 min; [4] 46.20–296.4 min; [8] 175–287 min; [9] and 119–259 min [10]). It is acknowledged that this comparison is not conclusive, since cadaveric trials omit some surgical aspects (i.e. intraoperative management of blood and the nervous system); however, it is reasonable to expect some implantation time reduction since 3D-printed plates eliminate the need for plate contouring and monitoring screw placement, and indeed, these reductions were directly observed in most previous studies using customised 3D-printed plates [3, 4]. Considering the cost of operating theatres in OECD countries is more than AU$2,000 per hour [11–13], these reductions in surgical time have the potential to deliver vital savings to the healthcare system. Shorter duration of surgery is also potentially beneficial to the patient, reducing rates of clinical complications.

While customised implants may reduce the surgical time, this comes as a trade-off to the additional engineering effort required to design and manufacture bespoke implants. The results from the current study show that the total time involving CT image processing, plate, screw and guide design for five out of the six customised plates was 3.0–9.5 h (Table 4). The plate design for Case 2, however, was considerably longer (20.2 h), which resulted from difficulties relating to an existing plate including metal artefact in the CT images and avoiding interference with the plates and screws. This challenging plate and the very first plate (Case 1, lateral) required three design iterations, which inevitably increased the design time. However, as the team gained greater experience, all plates designed after Case 2 required one or two design iterations, underscoring the importance of a consistent and knowledgeable design team to efficient customised plate design.

While the design of most fracture plates was achieved in slightly longer than a standard 8-h workday, for customised 3D-printed fracture plates to be used at scale these time frames likely need to be much shorter. Improvements may be achieved using software tools, such as an application that can rapidly place and adjust screw trajectories, as well as align 3D-printing templates for the screw heads. Moreover, tools that help assess implant surgical constraints, such as interference with anatomical structures or the limited surgical window used in Case 5 (Fig. 11), would help to maintain the knowledge of surgical constraints affecting the plate design. Hence, a dedicated software platform to better share and manage complex design information will help optimise this iterative design process, especially considering their frequent need to take place remotely.

The manufacturing of each plate required between 27.0 and 32.5 h to prepare the design, to 3D-print and for post-processing (Table 4). To a substantial extent, the manufacturing duration was dependent on the specific SLM printer; hence, with new SLM printer innovations such as bidirectional powder wipers or multiple more powerful lasers the build time will be reduced. A key issue with SLM printers is the high thermal gradients experienced, which for the current study required a stress relief anneal at 800 °C for 3 h under argon gas. Despite their poorer resolution, EBM printers impart less thermal stresses, potentially negating a need for the thermal treatment cycle. The post-processing for most of the plate designs involved simple hand tools, which accounted for approximately one hour of machining. However, for the Case 3 fracture plate the female threads for locking screws were machined using a multi-axis CNC which added a considerable amount of time. By developing software that can rapidly relay the design information to the CNC, these threads may be rapidly machined in a timely manner.

In terms of manufacturing accuracy, the RMS errors for the total surface of the plates (*RMS*_plate_all_) ranged from 0.13 to 0.47 mm, while the RMS error for the ventral surface facing the bone (*RMS*_plate_bone_) ranged from 0.10 to 0.49 mm (Table 2). This discrepancy falls either within the range reported for Ti6Al4V or pure titanium implants manufactured using powder bed fusion processes of 0.03–0.55 mm [14–20]. Nevertheless, since there are no standardised tolerances relating to the dimensional accuracy of standard fracture plates, it is unclear what surface accuracy is needed for customised fracture plates to achieve effective fixation. Standardised tolerances do exist for holes on standard fracture plates, where hole diameters may be exceeded by 0.1 mm to 0.2 mm and spherical tolerances may be exceeded by 0.05 mm to 0.075 mm [21, 22]; values that suggest the accuracy of customised plate manufacturing in the current and many previous studies require improvement. The errors observed in the current study were likely due to the relatively long length and thin cross-sections of the plates, a combination that gives rise to high thermal gradients that warp the component. While the heat treatment annealing used in the current study would have reduced thermally induced stresses and consequential warping, other approaches to reduce the extent or effects of thermal gradients may be investigated in future work, such as optimising the build orientation, improving the design of the support structure, or the use of other powder bed fusion techniques such as electron beam melting.

Possibly a more critical parameter related to manufacturing is the screw alignment accuracy, since misalignment poses a considerable risk of hip joint or soft tissue injury. Comparing the angulation of the pre-planned and implanted screws, the errors were considerably less for the Case 3 fracture plate (mean 2.77°, range 1.05°–6.34°; Table 2), which was the only plate to utilise CNC-machined threads for locking screws. For all other plates, *θ*_screw_ was mostly around 6° to 7° (range 0.73°–20.26°), which is similar to those reported for a patient-specific fracture plate used Merema et al. [5] (mean: 8.5°, range 0.4°–17.5) who notably utilised a 3D-printed alignment guide that was similar to the unused design for Case 4 (Fig. 7B). These results indicate that there are no considerable differences amongst the different alignment approaches trial (i.e. printed threads, polymer guides or hand taps) apart from the CNC-machining, which despite requiring increased resources provides a meaningful improvement.

The analysis of the plate positioning showed the misalignment ranged by translations and rotations of 1.74–13.00 mm and 1.58°–8.51°, respectively (Table 2). Each plate was positioned on the bone of the specimen by visual assessment and feel by the surgeon, a simple technique which has been previously used for customised plates [3, 5, 23]. However, this method becomes problematic with limited surgical exposure, especially for medial plates, where it was not possible to see the relative amount of contact between the bone and the plate. Further work using existing technologies is therefore needed to improve the positioning of personalised plates, including the use of surgical navigation, fluoroscopic imaging, robotics or more complex alignment guides [24] than those used in the current study.

A clinically relevant parameter of this study was the effectiveness of the customised fracture plate approach in reducing the acetabular fracture. This reduction was quantified by the differences between the planned positions of the bone fragments to those observed post-operatively, of which resultant translations and rotations ranged from 0.81–6.33 mm to 1.13°–19.39°, respectively (Table 2). The translation and rotation, respectively, correlated with the number of bone fragments (*p* = 0.013) and the fracture plate translation error (*p* = 0.018) (Table 3), meaning that more bone fragments or larger plate misalignment contributed to poorer fracture reduction. This tendency was apparent when planning the fracture reductions, where the initial positions of smaller fragments were difficult to identify. Moreover, many fragments were too small to be secured with a screw and could only be supported by buttressing the plate against their surface; an approach which was less controlled and likely to lead to poorer fracture reductions.

In many cases, the misalignments between the pre- and post-operative bone fragments exceeded the clinically accepted acetabular fracture reduction limit of 2 mm [25]. However, this parameter is computed for incongruities of the acetabular surface, which is a likely to have a different magnitude than the alignment of individual bone fragments that have a varying size, surface area and proximity to the acetabulum. Indeed, the pre-operative acetabulum, which had been virtually reduced to minimise incongruities, had an RMS deviation from the post-operative acetabulum that was acceptable for Cases 1 and 5 (1.74 mm and 1.38 mm, respectively), while unacceptable for the remaining cases (2.36–3.01 mm). Notably, the RMS deviation significantly correlated to the number of bone fragments (*p* = 0.026), suggesting that the severe acetabular fractures in the current study had contributed to the poor fracture reductions. Using the equation corresponding to this regression (Eq. (1)), the RMS deviation of the acetabular surface was below 2 mm for fractures with 5 or less fragments. This finding suggests that customised plates should not be used for acetabular fractures with more than 5 fragments; however, given the small number of specimens in the current study (*n* = 5), this finding ought to be confirmed with more fracture cases for it to be conclusive.

The generalisability of the current study may be limited by the number of specimens (*n* = 5) from donors that were generally older (age: 87.0 ± 11.9 years) than those undergoing this type of surgery (age: 75.5 ± 8.0 years [26]). The use of older donors led to lower bone densities than those of a younger cohort, making processing CT data more challenging, as well as weaker bone that generated many fragments of bone. These complications mean that the methods in the current study were mostly representative of more challenging acetabular fracture cases.

The use of fresh-frozen cadaveric specimens has its own limitations regarding the validity of the induced fractures, or the omission of some surgical aspects (i.e. intra-operative blood management, fluoroscopic confirmation of surgery, reduction). Consequently, the implantation times in the current surgery would be longer on patients in vivo; however, logically this increase would not be considerable. The use of cadaveric specimens did allow for very-high-dose post-operative CT imaging to determine surgical accuracy, which is not typically performed in vivo.

While the plates were implanted and felt secure, no mechanical testing was performed to evaluate the effectiveness of the fixation. Although the use of cadaveric specimens would have allowed for such tests, pelvis mechanical testing is a highly challenging area due to practical challenges relating to the large specimen size and management of the abdominal organs. Regardless, these mechanical tests were considered outside the aims of the current study, and ought to be explored in future work.

In summary, the implantation time of customised pelvic fracture plates in a cadaver series was lower compared to those reported in vivo using off-the-shelf plates. The time involved in designing the plates was approximately one day, while the manufacturing time ranged from 36.5 to 61.3 h. The longer manufacturing times related to CNC machining threads to accommodate locking screws; however, this type of fixation considerably improved the control of the screw trajectories and future work ought to focus on streamlining this manufacturing process for a customised design. The accuracy related to placing the fracture plates visually in the restricted surgical window of the pelvis was poor. Due to this misalignment, as well as the severity of the acetabular fractures, the reduction of the fractured acetabulum was unacceptable for some cases. These outcomes suggest that customised plates may be unsuitable for severe acetabular fractures (i.e. greater than five bone fragments), while the alignment of fracture plates ought to be improved by integrating existing technologies such as fluoroscopy or surgical navigation into the customised plate workflow. The times, accuracy and suggested improvements in the study may be used to guide future design and manufacturing workflows aimed at producing customised pelvic fracture plates for increased numbers of patients.

## Supplementary Information

**Additional file 1:** Finite element analysis of Case 1.
