# Supplementary material for: Fixation of pelvic acetabular fractures using 3D-printed fracture plates: a cadaver study
Source: J Orthop Surg Res. 2023 May 16;18:360. doi: 10.1186/s13018-023-03756-y (PMC10189937; doi:10.1186/s13018-023-03756-y)
Supplement: Supplementary file 1 — Additional file 1: Finite element analysis of Case 1. [file 13018_2023_3756_MOESM1_ESM.docx]

**Supplementary Material**

To evaluate whether the design of the customized plates provided sufficient strength, a finite element analysis was performed for Case 1, comprising the two fracture plates (Fig. 1). Using Hypermesh (version 2017.2; Altair Engineering, Michigan, USA), the hemipelvis fracture plates and screws were meshed with tetrahedral elements, while the femur was modelled with triangular shell elements (Table 1).

**
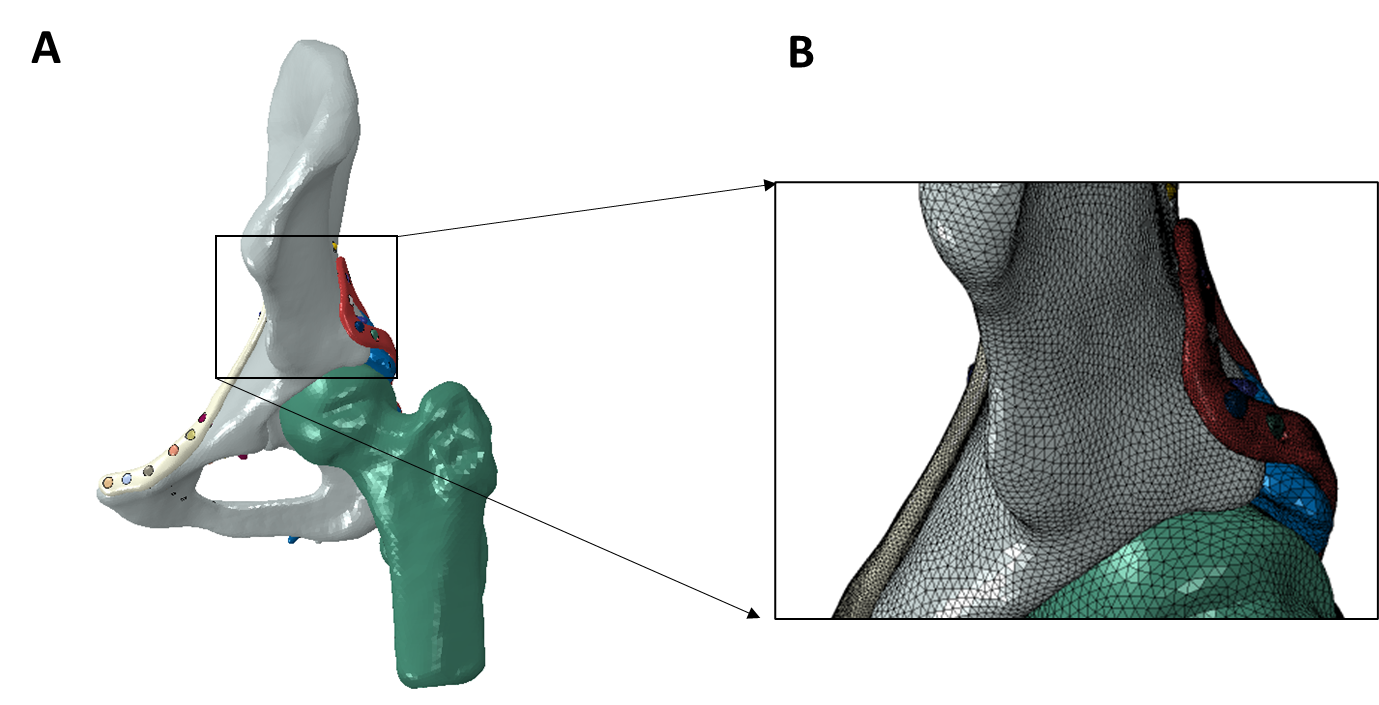
**

**Fig. 1:** Finite element models created for Case 1 (A), with a close up of the mesh indicated (B).

**Table 1**: Mesh characteristics

| Part | Element size, Mean±1.0SD (mm) | Number of elements |
| --- | --- | --- |
| Hemipelvis | 1.97±0.78 | 272,999 |
| Fragment | 0.68±0.25 | 10,360 |
| Medial fracture plate | 0.70±0.13 | 118,484 |
| Lateral fracture plate | 0.71±0.15 | 90,239 |
| Femur | 1.81±0.71 | 14,970 |
| Screws (26 in total) | 0.65±0.10 | 329,955 |

The shafts of the screws were converted to 3.5 mm cylinders, then a boolean operation was used to remove the bone necessary to accommodate each screw. The alignment between the hemipelvis and femur was set to those at contralateral toe-off during walking, which was 33.6° flexion, 8.1° adduction and 0° axial rotation [1]. The peak force for normal walking of 238 BW [2] was applied to the base of the femur, which for an average 70 kg female was 1634.3 N. The orientation of this force was set to 13° in the frontal plane and 31° in the transverse plane as outlined by Bergmann et al. [2]. The femur was held in this orientation by setting all rotations at its base to zero. The pelvis was clamped at the surface of the sacroiliac joint and the pubic symphysis. Hard contact was defined between the femoral head and the acetabular surface, and between contacting surfaces of the plate and bone. To model the effect of the locking screws, the screw head was tied to its contacting surface on the plate. The screw shafts were tied to the respective female surfaces of the bone.

The femur was set to a rigid body, while the pelvis modelled as an inhomogeneous linear elastic material, with moduli derived using a water-filled bone mineral density (BMD) phantom with densities of 250, 500, 750 and 1000 g/cm^3^ (Computerized Imaging Reference Systems, Norfolk, USA) that had been included in the CT scans. A linear regression was fitted to the greyscale values of these four known densities, which allowed the Hounsfield units in scan to be converted to apparent bone mineral density (ρ_app_) (Fig. 2). ρ_app_ was then converted to Young’s modulus, E, based on the power law for pelvic trabecular bone [3] (Eq. 1). The Poisson’s ratio for bone was set to a constant of 0.2 [3].

E= 2017.3ρ_app_^2.46^ (1)

The material properties of the plate and screws were modelled with properties of the Ti6Al4V grade 23 print material (Young’s modulus of 113.8 MPa and Poisson’s ratio of 0.342 [4]). A quasi-static simulation was applied with non-linear deformation, from which the von-Mises stresses of the metallic components was extracted.

**Fig. 2:** Regression between Hounsfield Units (HU) and apparent density (ρ_app_)

**Results**

A contour plot of the von-Mises stress of the medial and lateral plates is shown in Fig. 2. The Maximum stresses for the plate and screws (Table 2) were lower than the yield strength reported for Ti6Al4V alloys fabricated either by 3D printers with annealing (741-1045 MPa; [5]) or using traditional machining (790 MPa; [4]).

**Table 2**: Predicted stresses for plate and screws

| ID | Maximum von-Mises stress (MPa) | |
| --- | --- | --- |
|  | Plate | Screws |
| Case 1 medial | 169.2 | 539.5 |
| Case 1 lateral | 126.3 | 534.2 |


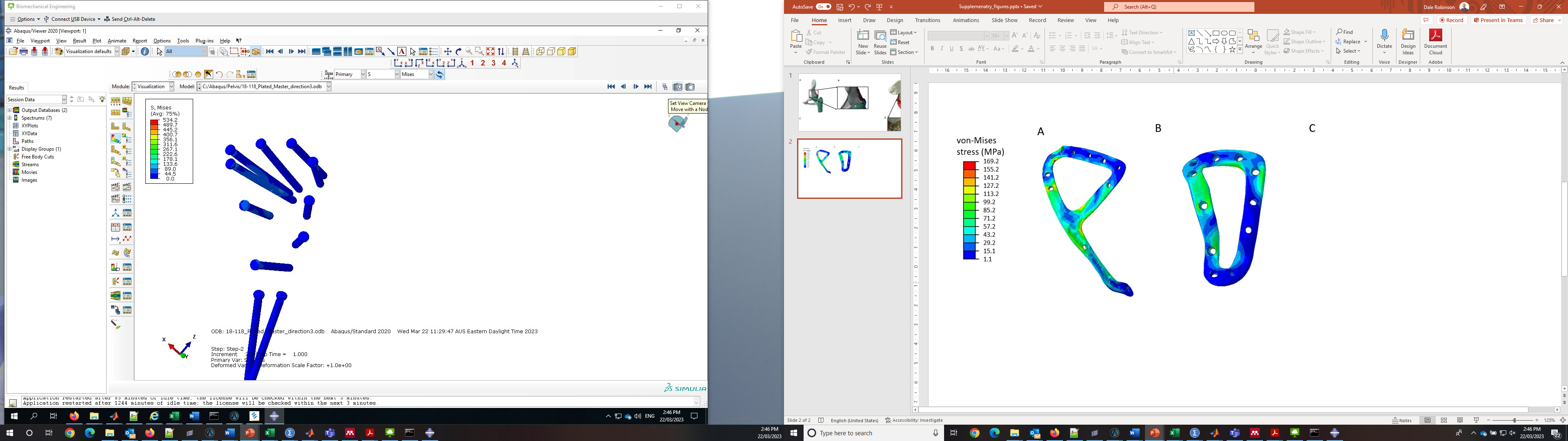


**Fig. 2:** Contour plot of von-Mises stress for the medial plate (A) and the lateral plate (B).

**References**

[1] K. M. Crossley, A. G. Schache, H. Ozturk, J. Lentzos, M. Munanto, and M. G. Pandy, “Pelvic and hip kinematics during walking in people with patellofemoral joint osteoarthritis compared to healthy age‐matched controls,” Arthritis Care & Research, 70(2): 309–314, 2018.

[2] G. Bergmann, G. Deuretzbacher, M. Heller, F. Graichen, and A. Rohlmann, “Hip contact and gait patterns from routine activities,” 34: 859–871, 2001.

[3] M. Dalstra, R. Huiskes, A. Odgaard, and L. van Erning, “Mechanical and textural properties of pelvic trabecular bone,” J Biomech,26(4-5): 523–535, 1993.

[4] MatWeb. "Titanium Ti-6Al-4V ELI (Grade 23), Annealed", 2023.

[5] S. Liu and Y. C. Shin, “Additive manufacturing of Ti6Al4V alloy: A review,” Mater Des, 164:107552, 2019.
